# The Impact of Marriage on Breastfeeding Duration: Examining the Disproportionate Effect of COVID-19 Pandemic on Low-Income Communities

**DOI:** 10.21203/rs.3.rs-5139881/v1

**Published:** 2024-12-17

**Authors:** Anna Charlotta Kihlstrom, Tara Stiller, Nishat Sultana, Grace Njau, Matthew Schmidt, Anastasia Stepanov, Andrew D. Williams

**Affiliations:** University of North Dakota; University of North Dakota; University of North Dakota; North Dakota Department of Health; North Dakota Department of Health; North Dakota Department of Health; University of North Dakota

**Keywords:** breastfeeding, marriage, COVID-19, education, income, American Indian, social support

## Abstract

**Background.:**

Marriage promotes breastfeeding duration through economic and social supports. The COVID-19 pandemic disproportionately affected marginalized communities and impacted women’s employment and interpersonal dynamics. This study examined how marital status affects breastfeeding duration across socioeconomic and racially minoritized groups during COVID-19, aiming to inform social support strategies for vulnerable families in public health crises.

**Methods.:**

Data were drawn from the 2017–2021 North Dakota Pregnancy Risk Assessment Monitoring System(weighted n=41433). Breastfeeding duration was self-reported, and 2-, 4-, and 6-month duration variables were calculated. Marital status(married, unmarried) and education (<high school education, ≥high school education) were drawn from birth certificates. Income(≤$48,000, >$48,000) and race/ethnicity (White, American Indian, Other) were self-reported. Infant birth date was used to identify pre-COVID(2017–2019) and COVID(2020–2021) births. Logistic regression estimated odds ratios and 95% confidence intervals for the association between marital status and breastfeeding duration outcomes. Models were fit overall, by COVID-19 era and by demographic factors. Lastly, demographic-specific models were further stratified by COVID era. Models were adjusted for maternal health and sociodemographic factors.

**Results.:**

Overall, married women consistently had 2-fold higher odds of breastfeeding across all durations during both pre-COVID and COVID eras. Pre-COVID, marriage was a stronger predictor for all breastfeeding durations in low-income women (4-month duration OR4.07,95%CI 2.52,6.58) than for high-income women (4-month duration OR1.76,95%CI 1.06,2.91). Conversely, during COVID, marriage was a stronger predictor of breastfeeding duration for high-income women (4-month duration OR 2.89,95%CI1.47,5.68) than low-income women (4-month duration OR 1.59,95%CI0.80, 3.15). Findings were similar among American Indian women and those with less than high school education, in that both groups lost the benefit of marriage on breastfeeding duration during the COVID-19 pandemic.

**Conclusion.:**

Marriage promotes breastfeeding duration, yet the benefit of marriage was reduced for low-socioeconomic and racially minoritized populations during the COVID-19 pandemic. Policies like paid parental leave and enhanced access to lactation consultants could help mitigate disproportionate impacts during public health crises. Continued research examining how major societal disruptions intersect with social determinants to shape breastfeeding outcomes can inform more equitable systems of care.

## Introduction

Exclusive breastfeeding for six months is recommended for optimal maternal and child health outcomes, such as lower rates of asthma and sudden infant death syndrome for infants and protection against cancer and type 2 diabetes for the mother (1). However, less than half of infants under six months old are exclusively breastfed (EBF) globally (2). In the United States (U.S.), while 84% of mothers initiate breastfeeding, only 47% exclusively breastfeed at three months and 25% exclusively breastfeed at six months (3).

Social support that encompasses emotional, informational and practical assistance, such as providing resources or hands-on help, from one’s social circle and healthcare professionals, promotes breastfeeding (4–7). Furthermore, partner support from male or same-sex partners may positively influence breastfeeding initiation, exclusivity and duration (8–12). For instance, verbal encouragement, support with household duties and caring for other children is supportive of EBF (13). Despite ample data on social and partner support, the association between marital status and breastfeeding remains underexamined. Previous studies suggest that married women have a higher prevalence of EBF at three months (1, 14) and six months (1, 15) compared to unmarried women. Marriage may support longer breastfeeding duration due to social and economic supports (16, 17). For example, a study of Illinois mothers (n = 103) enrolled in the Women, Infants, and Children (WIC) program found that married WIC participants were four times more likely to breastfeed for at least three months compared to single WIC participants (16).

The COVID-19 pandemic disrupted breastfeeding promotion practices in hospitals, including rooming-in and skin-to-skin contact after delivery (18–20). In the U.S., separating the maternal-infant dyad due to suspected or confirmed COVID-19 infection was associated with a decreased prevalence of breastfeeding initiation and duration (19). In a study of 85 COVID-19-positive mothers in New York City, 58% were separated from their newborns after birth (21). None of these separated mothers could start breastfeeding in the hospital, and only 12% breastfed at home (21). Conversely, 22% of non-separated mothers began breastfeeding in the hospital, and 28% continued at home (21). Similarly, a study from Italy found a decrease in EBF rates during the pandemic compared to pre-pandemic years: 69% of infants were EBF at hospital discharge during lockdown between March and May 2020 compared to 98% in 2018, and 32% were EBF at three months postpartum during the pandemic compared to 71% in 2018 (22). Furthermore, data suggest a reduction in in-person breastfeeding support from lactation consultants and medical providers during the pandemic, which may have negatively impacted breastfeeding practices (19, 23–25). For example, 45% of participants in a United Kingdom (U.K.) survey (n = 1365), reported not receiving enough breastfeeding support during COVID-19 lockdown (24).

Despite challenges to initiate breastfeeding during the pandemic, data suggest those who did initiate breastfeeding were able to breastfeed for longer durations, potentially linked to staying or working from home, and having less disruptions to breastfeeding (14, 26–28). An observational study in the U.S. found that once it was understood COVID-19 wasn’t significantly transmitted through breast milk, hospital policies allowing infants to stay with COVID-19-positive mothers promoted breastfeeding initiation and continuation at home (21). Notably, another U.S. study found the most gains in breastfeeding duration were among White and high-income women (28), aligning with a U.K. survey indicating that low-income and racialized minority mothers faced greater challenges in breastfeeding during pandemic lockdowns than White and high-income women (23). Remote work during the pandemic was more common among high-income, college-educated individuals (29), which may have contributed to the breastfeeding gains among high-income populations.

Given the importance of partner support in breastfeeding outcomes, the impact of COVID-19 on relationship dynamics merits further examination. Overall, married or cohabitating people had lower levels of distress during the pandemic compared to single people, except among racially minoritized married women who experienced more distress than other groups (30). Low-income married couples experienced more stress and lower marital satisfaction than those with a high socioeconomic status (SES) (31). A Romanian study suggests reduced marital satisfaction during lockdown for those with low SES, potentially due to economic hardship and loss of social connectedness (31). The implications of COVID-19 on women’s ability to work outside of the home may also have impacted family dynamics due to an increased workload at home and potential financial pressures. In the U.S., in September 2020 alone, approximately 865,000 women left the workforce compared to 216,000 men, often due to caregiving demands and limited remote work options (32, 33).

This study utilizes North Dakota Pregnancy Risk Assessment Monitoring System (ND PRAMS) data to explore the association between marital status and breastfeeding duration, and the potential modifying effects of race/ethnicity, income, and education in the context of COVID-19. To our knowledge, this is the first study that examines whether the beneficial effect of marital status on breastfeeding outcomes may have changed during the COVID-19 pandemic. The aim is to shed light on the types of health-promoting supports that are needed for low-income and racially minoritized families during and in the aftermath of public health crises.

## Methods

ND PRAMS is a collaborative effort between the North Dakota Department of Health and Human Services, and the Centers for Disease Control and Prevention (CDC). It is an ongoing, population-based surveillance system designed to monitor maternal behaviors, experiences, and outcomes before, during, and shortly after pregnancy. Survey data are supplemented with linked birth certificate data. The CDC developed PRAMS survey questions as part of a standard list from which states can select questions to include in their surveys. These questions have undergone testing and assessment among diverse populations as per CDC protocols. The survey methodology remains consistent across all racial/ethnic groups sampled (34).

There were 6934 individuals drawn from the 2017–2021 ND PRAMS. Participants were excluded from analysis if they had missing data on breastfeeding initiation (missing n=3433), marital status (missing n=0) and covariates (n missing = 708), for a final analytic sample of 2,793 individuals (weighted n= 41,433). Thus, each ND PRAMS participant represents approximately 14 women who recently gave birth in ND.

### Breastfeeding outcomes.

Breastfeeding initiation was self-reported response to the question “Did you ever breastfeed or pump breast milk to feed your new baby, even for a short period?” (yes/no). Breastfeeding duration was measured by analyzing maternal response to “Are you currently breastfeeding or feeding pumped milk to your new baby?” (yes/no) and “How many weeks or months did you breastfeed or feed pumped milk to your baby?” Participants reported breastfeeding duration in weeks or months, with weeks converted to months for analysis (two months = 4 weeks, four months = 16 weeks, six months = 24 weeks). The three duration variables were created (yes/no): two months, four months, six months.

### Marital status:

Marital status was drawn from birth certificate data and categorized as ‘married’ and ‘not married.’

### Race/Ethnicity:

ND PRAMS participants self-reported race/ethnicity: American Indian (AI; AI alone or biracial AI-white), White (women who self-reported as white alone) and women of Other Racial Identities (any other race/ethnicity including Black, Asian, Hispanic). Birth rates among the groups included in the racial/ethnic groups were too low during the study period (2017–2021) to inform sampling strata and specific racial/ethnic variables for analyses.

### Maternal education:

Education was drawn from birth certificate data and categorized as ≤High school and >High school.

### Income:

Income for the 12 months prior to pregnancy was self-reported and categorized as ≤$48,000 and > $48,000.

### COVID-19 Era:

Births occurring before year 2020 were identified as ‘Pre COVID-19,’ and births occurring during or after year 2020 were identified as ‘During COVID-19.’

Covariates were informed by literature (15–19,35): maternal age (<20, 20–35, >35), current insurance coverage (yes, no), preexisting chronic disease (depression, hypertension, diabetes; yes, no), pregnancy intention (wanted now, did not want now), Body Mass Index> 35 (yes, no), substance use prior to pregnancy (yes, no), ≥2 Adverse Childhood Experiences (yes, no), WIC enrollment (yes, no), infant sleeping alone (always, almost always, sometimes, rarely, never).

### Statistical Analysis

Descriptive statistics of weighted percent (and frequency) for all variables – overall and by marital status – were estimated using Rao Scott chi square. Descriptive statistics were also stratified by COVID-19 era.

A series of logistic regression models were fit to estimate odds ratios (OR) and 95% confidence intervals (95% CI) for the association between marital status and breastfeeding outcomes. In Model 1, an unadjusted model for the association between marital status and breastfeeding outcome was fit. For Model 2, maternal sociodemographic factors of age, race/ethnicity, education, income, insurance, WIC, pregnancy intention and ACEs were added to Model 1. Next, for Model 3, infant sleep and maternal health factors (chronic disease, obesity, and substance use prior to pregnancy) were added to Model 2.

To determine if the association between marital status and breastfeeding outcomes differed by COVID-19 era, we stratified Model 3 by COVID-19 era. Next, to determine if income, education, or race/ethnicity are potential effect modifiers in the association between marital status and breastfeeding outcomes, Model 3 was stratified by each demographic factor. Demographic-stratified models were fit for the overall sample, as well as by COVID-19 era. All models were fit for each breastfeeding duration outcome.

All statistical analyses were performed using SAS PROCSURVEY procedures in SAS 9.4 (SAS Institute Inc., Cary, NC, USA) to account for complex survey design and to apply sample weights to all analyses. This analysis was deemed exempt by the University of North Dakota Institutional Review Board.

## Results

Descriptive statistics were obtained for the overall sample (Table 1), and by COVID-19 era (Tables 2–3). For the overall sample (Table 1), breastfeeding initiation rates are comparable between married and unmarried women (98.5% vs. 96%, respectively), yet married women have higher rates of breastfeeding duration compared to non-married women (p<.01 for all duration comparisons). Married women were also more likely to have high income, education beyond high school, and identify as White.

During the pre-COVID-19 era (Table 2), breastfeeding initiation rates were 4 percent higher for married women compared to unmarried women (98.3% vs. 94.9%; p<.001). For breastfeeding duration pre-COVID-19, married women demonstrated higher rates at 2 months (83.1% vs. 59.7%; p < .001), 4 months (77.7% vs. 44.6%; p < .001), and 6 months (76.8% vs. 42.6%; p < .001).

During the COVID-19 pandemic (Table 3), there were no statistically significant differences in breastfeeding initiation rates between married and unmarried women. However, for breastfeeding duration, married women had higher rates at 2 months (86.1% vs. 64.2%; p < .001), 4 months (78% vs. 50%; p < .001), and 6 months (74.2% vs. 45.7%; p < .001).

Regression results for the association between marital status and breastfeeding outcomes are in Table 4.

In Model 1 for breastfeeding initiation, married women were significantly more likely to initiate breastfeeding (OR: 2.78, 95% CI: 1.55, 4.97). However, after adjusting for covariates in Model 2 and Model 3, this association became less pronounced (OR: 1.21, 95% CI: 0.54, 2.75; OR: 1.23, 95% CI: 0.54, 2.83, respectively). The findings are similar in the fully adjusted models Pre-COVID-19 (OR: 1.28, 95% CI: 0.49, 3.37) and during COVID-19 (OR: 1.02, 95% CI: 0.17, 6.16).

For duration outcomes, married women had about a two-fold higher risk for all time points. For example, in fully adjusted models, marital status was associated with two-fold higher odds of breastfeeding at four months (OR: 2.37, 95% CI: 1.82, 3.09). This association persisted through COVID-19-eras in the fully adjusted models for 4-month breastfeeding (Pre-COVID-19 OR: 2.75, 95% CI: 1.98, 3.82; During COVID-19 OR: 2.11, 95% CI: 1.29, 3.45).

Estimates for the association between marital status and breastfeeding outcomes among high- and low-income women, pre- and during COVID-19 are in Table 5. Marriage was a stronger predictor of all breastfeeding durations among low-income women overall and pre-COVID-19 compared to high-income women. For example, low income women had four-fold higher odds of breastfeeding at four months pre-COVID-19 than high-income women (OR: 4.07, 95% CI: 2.52, 6.58; OR: 1.76, 95% CI: 1.06, 2.91, respectively). However, during COVID-19, marriage became a stronger predictor of all breastfeeding durations at four months for high-income women (OR: 2.89, 95% CI 1.47, 5.68) compared to low-income women (OR: 1.59, 95% CI: 0.80, 3.15).

Table 6 includes regression results by education and COVID-19-era. Marriage was a stronger predictor of all breastfeeding durations among women with less than high school education overall and pre-COVID-19 compared to women with education beyond high school (4-month pre-COVID-19 OR: 4.17, 95% CI: 2.16, 8.08; OR: 2.01, 95% CI: 1.33, 3.05, respectively). Conversely, during COVID-19, marriage became a stronger predictor of breastfeeding for women with education beyond high school at four months (OR: 2.23, 95% CI:1.21, 4.12) compared to women with less than high school education (OR: 1.89, 95% CI: 0.85, 4.21).

Table 7 includes regression results by race/ethnicity and COVID-19-era. Marriage was a stronger predictor of nearly all breastfeeding durations overall and across COVID-19-eras among White women, who had two-fold higher odds of breastfeeding than unmarried White women. Among AI women, those who were married had approximately 70% higher odds of breastfeeding at all durations compared to unmarried AI women before COVID-19 (for example, 4-month OR: 1.70, 95% CI: 1.09, 2.67). However, the benefit of marriage on breastfeeding outcomes among AI women was lost during COVID-19 (for example, 4-month OR: 0.93, 95% CI: 0.44, 1.99).

## Discussion

This study explored the association between marital status and breastfeeding during COVID-19. Findings suggest socioeconomic status and racially minoritized status modify this association between marriage and breastfeeding. Pre-COVID-19, marriage more strongly predicted breastfeeding duration for low-income women. However, during COVID-19, low-income and AI women lost the breastfeeding benefit from marriage, while high-income and White women retained the benefit.

The benefit of marriage on breastfeeding was comparable pre- and during COVID-19 for two-month breastfeeding duration, however differences started to emerge at four and six months of breastfeeding duration. For instance, compared to pre-COVID-19 results, the odds of 4-month breastfeeding duration associated with marriage were 23% lower during the pandemic. This aligns with a 2023 study utilizing national PRAMS data (n = 118,139) which indicated no change in breastfeeding initiation, but an increase in early breastfeeding duration from 12.6 weeks pre-pandemic to 14.8 weeks during the pandemic, specifically among births in January and February 2020 (28). However, breastfeeding duration declined to pre-pandemic levels among the August 2020 birth cohort (28).

Emotional, tangible and financial supports in marriage may support longer breastfeeding duration (1, 14–16). However, the impact of specific types of support has underexamined (8). A 2020 integrative review of eight studies from Australia, Canada, Sweden, and the U.K. highlighted partner responsiveness as crucial for improved breastfeeding initiation, duration and exclusivity, emphasizing sensitivity to the mother’s needs and collaborative support (8). Similarly, a review of studies regarding breastfeeding barriers and supports emphasized the partner’s role in assisting with other children and household chores, and troubleshooting breastfeeding challenges (13). Furthermore, in a Canadian cross-sectional study (N = 76), women who perceived their partners as supportive of breastfeeding had greater confidence in their ability to breastfeed and produce milk (9). These findings underscore the multifaceted nature of support that partners can provide, and suggest that the quality of support, rather than marriage itself, plays a pivotal role in shaping breastfeeding outcomes. In the present study, the proportion of births to married people was consistently around 70% in the overall sample, and by COVID-19 era. This suggests that the changes in breastfeeding duration during the COVID-19 pandemic were likely not related to a change in the proportion of births to married people.

With workplace and school closures during COVID-19, parents were subject to layoffs or leaving the workforce to care for children. The burden of increased childcare and household responsibilities and job losses predominantly impacted women, resulting in what some scholars call a “shecession” (36, 37). In the U.S., in September 2020 alone, four times more women than men left the workforce to care for children, or due to limited remote work opportunities (32, 33). Married couples have the benefit of a second earner which may reduce the impact of job losses on household earnings (37). Still, the pandemic highlighted the lack of social safety nets in the U.S. compared to other wealthy nations, revealing stark gendered disparities in unpaid household labor and the absence of substantial paid parental leave and flexible work hours, as sociologist Jessica Calarco stated: “Other countries have social safety nets, the U.S. has women.” (38, 39).

In the present study, we observed disparities in breastfeeding duration among married women based on income, education and race. While the benefit of marriage was significantly reduced for low-income women during the pandemic, high-income women continued to see the benefit of marriage on breastfeeding duration. Women reported more mental distress than men during the pandemic (40, 41), and low-income couples were more prone to relationship strain (31), which may have affected the availability of marriage-related breastfeeding support. Furthermore, during the pandemic, those with a higher income were more likely to work from home (29), potentially allowing for more time to breastfeed compared to pre-pandemic times. This aligns with findings in a previous study based on PRAMS data before and during COVID-19 lockdowns, suggesting significant breastfeeding gains among high-income women during the pandemic (28). These gains were to a large extent attributed to the ability to work from or stay at home, suggesting that national paid family leave policies would improve breastfeeding rates (28). A study from Greece between January and December 2020 found that women not working at six months postpartum had nearly 12-times higher odds of EBF compared to employed women (14). Studies suggest that for each additional week of maternity leave, breastfeeding duration increases, with a more significant benefit when the leave is well-compensated (42–44). Correspondingly, countries with generously compensated parental leave from eight months to up to a year like Sweden and Finland, tend to have higher breastfeeding initiation and duration rates (42). In these countries, the ability for partners to uptake parental leave may also provide valuable support for mothers that promotes breastfeeding (42). In our study, the benefit of marriage for women with less than a high school education decreased during COVID-19 at four and six months, while those with higher education maintained pre-pandemic benefits. College-educated individuals had more work-from-home opportunities during COVID-19 (29), which may have positively influenced breastfeeding practices.

Regarding racial disparities, in ND, 54% of AI women initiate breastfeeding compared to 88.2% of White women (35). Within the context of colonization of AI people, federal policies in the twentieth century, such as the Save the Babies campaign, led to the displacement of traditional AI breastfeeding practices in favor of Western methods promoted by Euro-American women (35). Despite subsequent reversal of the Save the Babies campaign, the legacy of colonial policies persist, and the loss of cultural breastfeeding practices contribute to the lower breastfeeding rates among AI/AN populations (35, 45). Our observations suggest the COVID-19 pandemic exacerbated breastfeeding disparities among AI mothers, evidenced by a loss of benefit of marriage across breastfeeding outcomes.

The reasons for the loss of benefit of marriage among AI women is multifaceted. In part, it may be due to AI women’s predominant breadwinning role, with 64% contributing at least 40% of the household income (46), and COVID-19-related economic difficulties. Nearly 30% of AI women work in frontline occupations, for instance as home health aides and nursing assistants. On average, AI women make $0.60 cents to every dollar earned by White, non-Hispanic men (47). Even before the pandemic in 2019, AI mothers working full time were over five times more likely than White, non-Hispanic fathers to live below the poverty line (13.3% vs. 2.6%), with nearly 40% living below twice the poverty line (48). During the pandemic in early summer 2020, the rate of AI people working remotely was 8% lower than for White workers, partially due to their overrepresentation in frontline occupations, and underrepresentation in occupations that require a college degree and often enable remote work (49). With COVID-19-related school closures and lack of childcare, work exit rates during the pandemic were most significant for low SES women with school-aged children (50), likely further exacerbating the economic disparities experienced by AI women. These added stressors may have left little time and energy for AI women to breastfeed: according to recommendations on breastfeeding frequency, with feeds every 2–3 hours for 20–30 minutes each time, breastfeeding may take between 2–6 hours each day (35).

Moreover, AI communities were disproportionately impacted by the pandemic, with higher COVID-19-associated mortality rates than any other racial/ethnic group (51). In ND, AI individuals comprise 5% of the total population, yet by March 2022 they accounted for 9% of COVID-19 deaths in the state (52). Indigenous Elders play a crucial role in community wellbeing, including fostering resilience and healthy pregnancies (53). The loss of Elders may have devastating consequences on the transmission of traditional knowledge in Indigenous communities (54), including breastfeeding supports historically provided by Elders (55).

In preparation for future public health emergencies, it is essential to prioritize maternal and child health and increase financial and practical supports to low SES women. Barriers like unequal access to remote work, financial instability and loss of support networks hinder breastfeeding, particularly for low-income and AI women. Implementing policy-level changes such as expanding paid parental leave can promote breastfeeding and reduce breastfeeding disparities by allowing women to stay home longer without fear of financial loss, especially if they are in the breadwinning role. Furthermore, increasing practical and emotional supports for low SES and racially minoritized families during public health crises may help prevent breastfeeding disparities due to loss of at-home support. Referring qualifying expectant parents to programs such as the Nurse Family Partnership that has been successful in providing support, education and resources for low-income families for over four decades (56, 57), may help fill some of the gaps caused by loss of partner support. Furthermore, enhancing AI community access to Indigenous community health workers may deepen the cultural understanding of family dynamics during public health crises, while Indigenizing health care.

This study has several limitations. First, the cross-sectional study design limits causal interpretations. However, we were able to examine breastfeeding duration at three time periods, allowing us to understand how the effect of marriage may change during the postpartum period. Second, PRAMS only captures a binary yes/no response on breastfeeding initiation and lacks data on the exclusivity of breastfeeding or mixed feeding. Third, interpretations of findings within the “other” racial/ethnic category should be approached cautiously given the heterogeneity of this group. Last, self-reported data are susceptible to recall bias. Nonetheless, recall concerning events during the perinatal period is generally high, mitigating concerns about recall bias (58).

This study has several notable strengths. First, to the best of our knowledge, this was the first study to examine the effect of marriage on breastfeeding before and during the COVID-19 pandemic, providing data on how social aspects of the pandemic, like gender roles and social supports, influenced health outcomes. Second, our research contributes to the expanding body of literature on the varied effects of the COVID-19 pandemic on marginalized communities in the U.S. For instance, ND PRAMS employs a weighted design that oversampled AI women, ensuring that the analysis provided a more accurate representation of pregnant women in ND. The increased inclusion of AI women is crucial for addressing maternal and infant health disparities and centering AI culture. Third, availability of robust social determinants of health (such as adverse childhood experiences, pregnancy intention and infant sleep behaviors) in ND PRAMS allowed us to include a robust set of confounders in analyses.

## Conclusion

This study highlights how the COVID-19 pandemic exacerbated existing disparities in breastfeeding duration, such that during the COVID-19 pandemic, low-SES women and AI women lost the breastfeeding benefit of marriage. These findings underscore the need for strengthening social safety nets and providing more robust supports to vulnerable populations during public health emergencies. Policies like paid parental leave, enhanced access to lactation consultants, and Indigenizing maternal health services could help mitigate disparities and promoting equitable breastfeeding practices. Continued research examining how major societal disruptions intersect with social determinants to shape breastfeeding outcomes can inform more inclusive systems of care.

## Figures and Tables

**Table 1.** Distribution of breastfeeding outcomes and covariates, overall and my marital status (weighted n=41,433)

	Overall (weighted n=41433)	Married (weighted n=28991)	Not married (weighted n=12442)	p-value
**BF initiation**				*p*<.001
Yes	97.8 (40,511)	98.5 (28564)	96 (11,947)	
No	2.2 (922)	1.5 (427)	4 (495)	
**2 Month BF**				*p*<.001
Yes	77.2 (31,990)	84.1 (24,382)	61.2 (7,609)	
No	22.8 (9,443)	15.9 (4,609)	38.8 (4,834)	
**4 month BF**				*p*<.001
Yes	68.4 (28,318)	77.8 (22,552)	46.3 (5,767)	
No	31.6 (13,115)	22.2 (6,439)	53.7 (6,676)	
**6 month BF**				*p*<.001
Yes	66.2 (27,441)	75.9 (22,014)	43.6 (5,426)	
No	33.8 (13,993)	24.1 (6,976)	56.4 (7,017)	
**Income**				*p*<.001
>$48,000	66.3 (27,463)	81.5 (23,618)	30.9 (3,845)	
≤$48,000	33.7 (13,970)	18.5 (5,372)	69.1 (8,598)	
**Education**				*p*<.001
<High school	26.2 (10,856)	16.1 (4,661)	49.8 (6,195)	
>High school	73.8 (30,577)	83.9 (24,329)	50.2 (6,248)	
**Race**				*p*<.001
AI	6.3 (2,615)	22.1 (577)	77.9 (2,038)	
Other	11.9 (4,924)	65.2 (3,211)	34.8 (1,713)	
White	81.8 (33,895)	74.4 (25,203)	25.6 (8,692)	
**Age**				*p*<.001
<20 years	2.9 (1,211)	9 (109)	91 (1,102)	
20–35	83.4 (34,567)	70.3 (24,303)	29.7 (10,263)	
35+	13.7 (5,656)	80.9 (4,578)	19.1 (1,078)	
**Insurance**				*p*<.001
Yes	93.9 (41,433)	96.8 (28,071)	87.1 (10,832)	
No	6.1 (2,531)	3.2 (919)	12.9 (1,611)	
**Chronic disease**				*p*<.001
Yes	21.4 (8,886)	16.5 (4,780)	33 (4,105)	
No	78.6 (32,548)	83.5 (24,210)	67 (8,338)	
**Pregnancy intention**				*p*<.001
Wanted now	95.7 (39,644)	97.2 (28,189)	92.1 (11,455)	
Did not want now	4.3 (1,789)	2.8 (801)	7.9 (988)	
**Body Mass Index**				*p*<.05
≥35	60.7 (25,137)	59.2 (17,152)	64.2 (7,985)	
<35	39.3 (16,297)	40.8 (11,838)	35.8 (4,458)	
**Substance use prior to pregnancy**				*p*>.05
Yes	75.2 (31,139)	75.2 (31,139)	76.9 (9,573)	
No	24.8 (10,294)	24.8 (10,294)	23.1 (2,870)	
**≥2 ACEs**				*p*<.001
Yes	20.4 (8,449)	14 (4,053)	35.3 (4,396)	
No	79.6 (32,984)	86 (24,937)	64.7 (8,047)	
**WIC**				*p*<.001
Yes	80.7 (33,449)	90.1 (26,126)	58.9 (7,323)	
No	19.3 (7,985)	9.9 (2,865)	41.1 (5,120)	
**Infant sleep own bed**				*p*<.001
Always	59.3 (24,571)	62.9 (18,233)	50.9 (6,338)	
Almost always	17.7 (7,327)	17.8 (5,169)	17.3 (2,158)	
Sometimes	9.6 (3,985)	8.8 (2,553)	11.5 (1,431)	
Rarely	5.1 (2,104)	3.6 (1,043)	8.5 (1,061)	
Never	8.3 (3,446)	6.9 (1,992)	11.7 (1,454)	

**Table 2:** Distribution of breastfeeding outcomes and covariates, overall and my marital status, before COVID-19, 2017–2019

	Overall (weighted n=27625)	Married (weighted n=19293)	Not married (weighted n=8332)	p-value
**BF initiation**				
Yes	97.2 (26,863)	98.3 (18,957)	94.9 (7,906)	*p*<.001
No	2.8 (762)	1.7 (336)	5.1 (426)	
**2 Month BF**				*p*<.01
Yes	76 (21,004)	83.1 (16,033)	59.7 (4,971)	
No	24 (6,620)	16.9 (3,259)	40.3 (3,361)	
**4 month BF**				*p*<.001
Yes	67.7 (18,702)	77.7 (14,989)	44.6 (3,713)	
No	32.3 (8,923)	22.3 (4,303)	55.4 (4,619)	
**6 month BF**				*p*<.001
Yes	66.5 (18,367)	76.8 (14,818)	42.6 (3,549)	
No	33.5 (9,258)	23.2 (4,474)	57.4 (4,783)	
**Income**				*p*<.001
>$48,000	64.4 (17,797)	80.7 (15,573)	26.7 (2,224)	
≤$48,000	35.6 (9,828)	19.3 (3,720)	73.3 (6,108)	
**Education**				*p*<.001
<High school	25.9 (7,168)	15.6 (3,012)	49.9 (4,155)	
>High school	74.1(20,457)	84.4 (16,281)	50.1 (4,176)	
**Race**				*p*<.001
AI	6.9 (1,894)	1.9 (375)	18.2 (1,519)	
Other	11.1 (3,075)	10.7 (2,070)	12.1 (1,006)	
White	82 (22,655)	87.3 (16,848)	69.7 (5,807)	
**Age**				*p*<.001
<20 years	2.9 (788)	0.4 (71)	8.6 (717)	
20–35	84 (23,224)	84.5 (16,311)	83 (6,912)	
35+	13.1 (3,613)	15.1 (2,910)	8.4 (703)	
**Insurance**				*p*<.001
Yes	93.5 (25,826)	96.8 (18,666)	85.9 (7,160)	
No	6.5(1,799)	3.2 (627)	14.1 (1,172)	
**Chronic disease**				*p*<.001
Yes	19.7 (5,433)	14.6 (2,814)	31.4 (2,619)	
No	80.3 (22,192)	85.4 (16,479)	68.6 (5,713)	
**Pregnancy intention**				*p*<.001
Wanted now	95.2 (26,292)	96.6 (18,634)	91.9 (7,657)	
Did not want now	4.8 (1,333)	3.4 (658)	8.1 (675)	
**Body Mass Index**				*p*>.05
≥35	59.7 (16,493)	58.1 (11,208)	63.4 (5,284)	
<35	40.3 (11,132)	41.9 (8,084)	36.6 (3,048)	
**Substance use prior to pregnancy**				*p*>.05
Yes	74.9 (20,704)	74.3 (14,340)	76.4 (6,364)	
No	25.1 (6,921)	25.7 (4,952)	23.6 (1,968)	
**≥2 ACEs**				*p*<.001
Yes	20.3 (5,598)	14.2 (2,734)	34.4 (2,863)	
No	79.7 (22,027)	85.8 (16,558)	65.6 (5,468)	
**WIC**				*p*<.001
Yes	78.9 (21,808)	89 (17,180)	55.5 (4,627)	
No	21.1 (5,817)	11 (2,113)	44.5 (3,705)	
**Infant sleep own bed**				*p*<.001
Always	60.3 (16,658)	63 (12,149)	54.1 (4,510)	
Almost always	16.9 (4,663)	17.8 (3,426)	14.8 (1,237)	
Sometimes	8.9 (2,471)	8 (1,551)	11 (920)	
Rarely	5.5 (1,506)	3.7 (727)	9.3 (779)	
Never	8.4(2,326)	7.5 (1,440)	10.6 (886)	

**Table 3:** Distribution of breastfeeding outcomes and covariates, overall and my marital status, during the COVID-19-era 2020–2021

	Overall (weighted n=13808)	Married (weighted n=28991)	Not married (weighted n=12442)	p-value
**BF initiation**				*p*>.05
Yes	98.8 (13,649)	98.5 (9697)	98.3 (3952)	
No	1.2 (159)	1.5 (90)	1.7 (69)	
**2 Month BF**				*p*<.001
Yes	79.6 (10,986)	86.1 (8,348)	64.2 (2,638)	
No	20.4 (2,822)	13.9 (1,349)	35.8 (1,473)	
**4 Month BF**				*p*<.001
Yes	69.6 (9,616)	78 (7,562)	50 (2,054)	
No	30.4 (4,192)	22 (2,135)	50 (5,057)	
**6 Month BF**				*p*<.001
Yes	65.7 (9,074)	74.2 (7,196)	45.7 (1,878)	
No	34.3 (4,735)	25.8 (2,502)	54.3 (2,233)	
**Income**				*p*<.001
>$48,000	70 (9,667)	83 (8,046)	39.4 (1,621)	
≤$48,000	30 (4,142)	17 (1,652)	60.6 (2,490)	
**Education**				*p*<.001
<High school	26.7 (3,688)	17 (1,649)	49.6 (2,040)	
>High school	73.3 (10,120)	83 (8,049)	50.4 (2,072)	
**Race**				*p*<.001
AI	5.2 (721)	2 (201)	12.6 (519)	
Other	13.4 (1,848)	11.8 (1,141)	17.2 (707)	
White	81.4 (11,240)	86.2 (8,355)	70.2 (2,885)	
**Age**				*p*<.001
<20 years	3.1 (423)	0.4 (38)	9.4 (385)	
20–35	82.1 (11,343)	82.4 (7,992)	81.5 (3,351)	
35+	14.8 (2,043)	17.2 (1,668)	9.1 (375)	
**Insurance**				*p*<.001
Yes	94.7 (13,077)	97 (9,406)	89.3 (3,672)	
No	5.3 (731)	3 (292)	10.7 (439)	
**Chronic disease**				*p*<.001
Yes	25 (3,453)	20.3 (1,966)	36 (1,486)	
No	75 (10,356)	79.7 (7,731)	64 (2,625)	
**Pregnancy intention**				*p*<.001
Wanted now	96.7 (13,353)	98.5 (9,555)	92.4 (3,798)	
Did not want now	3.3 (456)	1.5 (143)	7.6 (313)	
**Body Mass Index**				*p*>.05
≥35	62.6 (8,644)	61.3 (5,943)	65.7 (2,701)	
<35	37.4 (5,164)	38.7 (3,754)	34.3 (1,410)	
**Substance use prior to pregnancy**				*p*>.05
Yes	75.6 (10,435)	74.5 (7,226)	78.1 (3,210)	
No	24.4 (3,373)	25.5 (2,472)	21.9 (901)	
**≥2 ACEs**				*p*<.001
Yes	20.7 (2,852)	13.6 (1,319)	37.3 (1,533)	
No	79.3 (10,957)	86.4 (8,379)	62.7 (2,578)	
**WIC**				*p*<.001
Yes	84.3 (11,641)	92.2 (8,945)	65.6 (2,695)	
No	15.7 (2,168)	7.8 (752)	34.4 (1,416)	
**Infant sleep own bed**				*p*<.001
Always	57.3 (7,913)	62.7 (6,084)	44.5 (1,829)	
Almost always	19.3 (2,664)	18 (1,743)	22.4 (921)	
Sometimes	11 (1,514)	10.3 (1,002)	12.4 (511)	
Rarely	4.3 (598)	3.3 (316)	6.9 (283)	
Never	8.1 (1,120)	5.7 (552)	13.8 (567)	

**Table 4:** Crude and adjusted models for association between marital status and breastfeeding, pre and during COVID-19 (weighted n=41,433)

	Model 1 OR (95%CI)	Model 2^a^ OR (95%CI)	Model 3^b^ OR (95%CI)	Fully Adjusted Pre-COVID-19^b^ OR (95%CI)	Fully adjusted COVID-19^b^ OR (95%CI)
**BF Initiation**					
Marital Status					
Yes	2.78 (1.55, 4.97)	1.21 (0.54, 2.75)	1.23 (0.54, 2.83)	1.28 (0.49, 3.37)	1.02 (0.17, 6.16)
No	Ref	Ref	Ref	Ref	Ref
Income					
High	-	0.93 (0.44, 1.97)	0.90 (0.43, 1.89)	1.18 (0.50, 2.80)	0.23 (0.07, 0.80)
Low	-	Ref	Ref	Ref	Ref
Education	-				
<high school	-	0.20 (0.11, 0.39)	0.21 (0.11, 0.41)	0.23 (0.11, 0.48)	0.12 (0.03, 0.52)
>high school	-	Ref	Ref	Ref	Ref
Race	-				
AI	-	0.29 (0.16, 0.55)	0.28 (0.15, 0.53)	0.35 (0.17, 0.70)	0.13 (0.03, 0.51)
Other	-	0.73 (0.27, 1.99)	0.74 (0.28, 1.96)	0.72 (0.23, 2.31)	1.12 (0.24, 5.31)
White	-	Ref	Ref	Ref	Ref
**2-month BF duration**					
Marital Status					
Yes	3.36 (2.69, 4.19)	2.17 (1.64, 2.87)	2.15 (1.61, 2.86)	2.14 (1.51, 3.04)	2.39 (1.41, 4.03)
No	Ref	Ref	Ref	Ref	Ref
Income	-				
>$48,000	-	1.20 (0.89, 1.61)	1.24 (0.91, 1.67)	1.28 (0.89, 1.85)	1.22 (0.70, 2.12)
≤$48,000	-	Ref	Ref	Ref	Ref
Education	-				
<high school		0.70 (0.53, 0.93)	0.73 (0.55, 0.98)	0.76 (0.54, 1.07)	0.63 (0.38, 1.07)
>high school	-	Ref	Ref	Ref	Ref
Race	-				
AI	-	0.70 (0.54, 0.90)	0.72 (0.56, 0.93)	0.67 (0.50, 0.90)	0.71 (0.42, 1.21)
Other	-	1.36 (0.94, 1.97)	1.28 (0.87, 1.87)	1.06 (0.63, 1.78)	1.42 (0.79, 2.57)
White	-	Ref	Ref	Ref	Ref
**4-month BF duration**					
Marital Status					
Yes	4.06 (3.29, 5.00)	2.38 (1.84, 3.09)	2.37 (1.82, 3.09)	2.75 (1.98, 3.82)	2.11 (1.29, 3.45)
No	Ref	Ref	Ref	Ref	Ref
Income	-				
>$48,000	-	1.48 (1.12, 1.95)	1.54 (1.17, 2.02)	1.49 (1.06, 2.08)	1.80 (1.09, 2.97)
≤$48,000	-	Ref	Ref	Ref	Ref
Education	-				
<high school	-	0.65 (0.50, 0.84)	0.68 (0.52, 0.88)	0.66 (0.48, 0.91)	0.67 (0.42, 1.08)
>high school	-	Ref	Ref	Ref	Ref
Race					
AI	-	0.68 (0.54, 0.87)	0.71 (0.56, 0.91)	0.65 (0.48, 0.86)	0.73 (0.44, 1.22)
Other	-	1.26 (0.91, 1.76)	1.20 (0.86, 1.68)	0.96 (0.60, 1.53)	1.31 (0.77, 2.24)
White	-	Ref	Ref	Ref	Ref
**6-month BF duration**					
Marital Status					
Yes	4.08 (3.32, 5.02)	2.32 (1.79, 2.99)	2.31 (1.78, 3.00)	2.77 (1.99, 3.85)	1.87 (1.15, 3.03)
No	Ref	Ref	Ref	Ref	Ref
Income	-				
>$48,000	-	1.51 (1.15, 1.98)	1.58 (1.21, 2.08)	1.62 (1.16, 2.27)	1.72 (1.05, 2.80)
≤$48,000	-	Ref	Ref	Ref	Ref
Education	-				
<high school	-	0.61 (0.47, 0.79)	0.64 (0.49, 0.83)	0.68 (0.50, 0.94)	0.52 (0.33, 0.84)
>high school	-	Ref	Ref	Ref	Ref
Race	-				
AI	-	0.66 (0.52, 0.83)	0.69 (0.54, 0.89)	0.64 (0.47, 0.85)	0.69 (0.42, 1.13)
Other	-	1.12 (0.81, 1.55)	1.06 (0.76, 1.47)	0.94 (0.59, 1.49)	1.05 (0.62, 1.79)
White	-	Ref	Ref	Ref	Ref

a) Variables accounted for in model 2: age, maternal race/ethnicity, education, income, insurance, WIC, pregnancy intention, ACEs.

b) Variables accounted for in model 3: chronic health problems, obesity, substance use before pregnancy

**Table 5:** Association between marital status and breastfeeding outcomes, stratified by income, overall and by COVID-19 era. (weighted n=41,433)

	Overall		Pre-COVID-19		COVID-19	
Breastfeeding outcome	Low Income OR (95%CI)	High Income OR (95%CI)	Low Income OR (95%CI)	High Income OR (95%CI)	Low Income OR (95%CI)	High Income OR (95%CI)
**BF Initiation**	1.18 (0.38, 3.70)	1.57 (0.39, 6.39)	1.18 (0.34, 4.13)	1.61 (0.28, 9.46)	1.05 (0.33, 3.29)	1.23 (0.08, 18.5)
**2-month BF duration**	2.57 (1.69, 3.92)	1.99 (1.29, 2.07)	2.73 (1.64, 4.52)	1.64 (0.93, 2.86)	2.29 (1.05, 4.99)	2.71 (1.33, 5.54)
**4-month BF duration**	2.93 (1.99, 4.32)	2.15 (1.44, 3.20)	4.07 (2.52, 6.58)	1.76 (1.06, 2.91)	1.59 (0.80, 3.15)	2.89 (1.47, 5.68)
**6-month BF duration**	2.74 (1.86, 4.03)	2.16 (1.46, 3.20)	3.90 (2.42, 6.28)	1.86 (1.13, 3.05	1.27 (0.64, 2.52)	2.64 (1.37, 5.10)

**Table 6.** Association between marital status and breastfeeding outcomes, stratified by education, overall and by COVID-19-era. (weighted n=41,433)

	Overall		Pre-COVID-19		COVID-19	
Breastfeeding outcome	Less than high school OR (95%CI)	More than high school OR (95%CI)	Less than high school OR (95%CI)	More than high school OR (95%CI)	Less than high school OR (95%CI)	More than high school OR (95%CI)
**BF Initiation**	0.98 (0.35, 2.77)	2.30 (0.63, 8.32)	0.86 (0.31, 2.36)	1.36 (0.41, 4.53)	1.35 (0.20, 8.98)	5.96 (0.79, 45.17)
**2-month BF duration**	2.34 (1.41, 3.89)	2.08 (1.45, 2.98)	2.19 (1.15, 4.17)	1.95 (1.25, 3.05)	3.08 (1.15, 8.26)	2.28 (1.22, 4.26)
**4-month BF duration**	3.20 (1.97, 5.18)	2.12 (1.51, 2.98)	4.17 (2.16, 8.08)	2.01 (1.33, 3.05)	1.89 (0.85, 4.21)	2.23 (1.21, 4.12)
**6-month BF duration**	2.87 (1.78, 4.60)	2.15 (1.54, 3.01)	4.24 (2.18, 8.23)	2.06 (1.36, 3.13)	1.58 (0.75, 3.35)	2.14 (1.17, 3.91)

**Table 7:** Association between marital status and breastfeeding outcomes, stratified by race, overall and by COVID-19-era. (weighted n=41,433)

	Overall			Pre-COVID-19			COVID-19		
	White OR (95%CI)	American Indian OR (95%CI)	Other OR (95%CI)	White OR (95%CI)	American Indian OR (95%CI)	Other OR (95%CI)	White OR (95%CI)	American Indian OR (95%CI)	Other OR (95%CI)
**BF Initiation**	1.25 (0.37, 4.27)	2.71 (0.97, 7.61)	1.95 (0.46, 8.21)						
**2-month BF duration**	2.30 (1.62, 3.26)	1.62 (1.11, 2.36)	2.11 (1.01, 4.43)	2.29 (1.49, 3.52)	1.76 (1.12, 2.73)	1.39 (0.50, 3.89)	2.16 (1.14, 4.08)	1.26 (0.59, 2.70)	4.37 (1.53, 12.48)
**4-month BF duration**	2.57 (1.85, 3.57)	1.48 (1.01, 2.16)	2.28 (1.21, 4.33)	2.77 (1.84, 4.17)	1.70 (1.09, 2.67)	1.88 (0.73, 4.87)	2.22 (1.23, 4.00)	0.93 (0.44, 1.99)	2.51 (0.91, 6.87)
**6-month BF duration**	2.51 (1.81, 3.48)	1.57 (1.08, 2.28)	2.08 (1.11, 3.91)	2.82 (1.87, 4.25)	1.78 (1.13, 2.79)	1.94 (0.75, 5.05)	2.01 (1.13, 3.57)	0.94 (0.41, 2.15)	1.76 (0.66, 4.70)

## Availability of data and materials:

North Dakota PRAMS data are available upon request and approval from the North Dakota Department of Health and Human Services. For more information please visit www.hhs.nd.gov/pregnancy-risk-assessment-monitoring-system-prams or email prams@nd.gov.

## Abbreviations

95% CI: 95% Confidence Intervals
ACEs: Adverse Childhood Experiences
AI: American Indian
CDC: Centers for Disease Control and Prevention
EBF: Exclusively Breastfed
ND: North Dakota
OR: Odds ratio
PRAMS: Pregnancy Risk Assessment Monitoring System
SES: socioeconomic status
U.K.: United Kingdom
U.S.: United States
WIC: Women, Infants, Children program
